# CC Chemokine 2 Promotes Ovarian Cancer Progression through the MEK/ERK/MAP3K19 Signaling Pathway

**DOI:** 10.3390/ijms241310652

**Published:** 2023-06-26

**Authors:** Wei Liu, Lei Wang, Jiajia Zhang, Kun Cheng, Wenming Zheng, Zhenling Ma

**Affiliations:** College of Life Sciences, Henan Agricultural University, Zhengzhou 450002, China

**Keywords:** ovarian cancer, CC-chemokine ligand 2, tumor progression, MAPK/ERK pathway, mitogen-activated protein three kinase 19

## Abstract

Ovarian cancer is a gynecological tumor with an incidence rate lower than those of other gynecological tumor types and the second-highest death rate. CC chemokine 2 (CCL2) is a multifunctional factor associated with the progression of numerous cancers. However, the effect of CCL2 on ovarian cancer progression is unclear. Here, we found that exogenous CCL2 and the overexpression of CCL2 promoted the proliferation and metastasis of ovarian cancer cells. On the other hand, CCL2 knockdown via CRISPR/Cas9 inhibited ovarian cancer cell proliferation, migration, and invasion. The present study demonstrated that mitogen-activated protein three kinase 19 (MAP3K19) was the key CCL2 target for regulating ovarian cancer progression through transcriptome sequencing. Additionally, MAP3K19 knockout inhibited ovarian cancer cell proliferation, migration, and invasion. Furthermore, CCL2 increased MAP3K19 expression by activating the mitogen-activated protein kinase kinase (MEK)/extracellular signal-regulated kinase (ERK) pathway. The present study showed the correlation between CCL2 and ovarian cancer, suggesting that CCL2 may be a novel target for ovarian cancer therapy.

## 1. Introduction

CC chemokine 2 (CCL2), also called monocyte chemotactic protein-1 (MCP-1), is the first CC chemokine identified in humans to attract monocytes and immune cells chemotactically [1,2]. Human CCL2 comprising 76 amino acids has a molecular size of 13 kDa. CCL2 is located on chromosome 17 (chr.17, q11.2). CCL2 was originally called a tumor-derived chemokine because it was discovered in tumor cells in vitro [3]. However, CCL2 is secreted by several cell types, such as epithelial cells, fibroblasts, and T cells [4,5,6,7]. Cytokine receptors have been recognized as G-protein-coupled receptors that are seven-transmembrane receptors. The activated G-protein-coupled receptors (GPCRs) are responsible for cAMP, diacylglycerol (DAG), and inositol 1,4,5-trisphate (IP3) formation and protein A and C activation. CCL2 mainly mediates its functions by binding to its receptor, namely, chemokines (C-C motif) receptor 2 (CCR2), triggering a series of signal transduction reactions [8]. The CCL2–CCR2 signaling axis is crucial for cancer progression. Studies showed that CCL2–CCR2 axis inactivation may result in an antitumor function in a malignant glioma model [9].

Ovarian cancer is a malignant tumor with an incidence rate lower than that of other gynecological tumors but the second-highest death rate [10,11]. High-grade serous ovarian cancer (HGSOC) is the fifth most common cancer among women worldwide and the leading cause of gynecologic malignancy death in the United States [12]. Studies showed that GATA binding protein 3 (GATA3) is highly expressed in HGSOC. GATA3 is associated with poor prognosis in patients with HGSOC and is a promising therapeutic target [13]. Ovarian cancer is often diagnosed in late stages because of the lack of symptoms in the early stages and the availability of limited screening and diagnosis methods, resulting in poor treatment outcomes [14,15,16,17]. Cytokines have numerous biological functions, such as cell growth regulation, cell differentiation and maturation, immune response, participation in the inflammatory response, wound healing, and tumor growth. Cytokines can mediate the interaction between immune cells and ovarian cancer cells and are vital for ovarian cancer development. Studies demonstrated that epithelial ovarian cancer growth and progression are promoted by the tumor necrosis factor a, interleukin 1β, and interleukin 6 produced by activated innate immune cells [18,19,20,21]. Studies demonstrated that cytokine antagonists might serve as promising therapeutic targets. Studies showed that plasma CCL2 levels are elevated in patients with high-grade ovarian cancer compared with healthy volunteers [22]. Additionally, CCL2 expression is upregulated in MA-148 (an ovarian cancer cell line) under treatment with paclitaxel and/or carboplatin [23]. Our previous study showed that recombinant CCL2 protein promoted ovarian cancer cell proliferation by activating the ERK pathway [24]. Furukawa et al. exhibited that exogenous CCL2 stimulated SKOV-3 cell migration and adhesion, which could be attenuated by adding a CCR2 antagonist [25]. Additionally, CCL2 inhibition by neutralizing antibodies reduced epithelial ovarian cancer cell invasion, indicating the crucial role of CCL2 in ovarian cancer development [26]. However, the mechanism of action of CCL2 in ovarian cancer development remains unclear.

We hypothesized that CCL2 plays an important role in ovarian cancer progression. The present study evaluated the vital role of CCL2 in ovarian cancer and explored the mechanism of CCL2 in the development of ovarian cancer. Exogenous CCL2 and the overexpression of CCL2 promoted the proliferation, migration, and invasion of ovarian cancer cells, whereas CCL2 inhibition decreased the biological functions of ovarian cancer cells. Additionally, mitogen-activated protein three kinase 19 (MAP3K19) was the key target for CCL2 to regulate ovarian cancer progression. MAP3K19 knockout inhibited ovarian cancer cell proliferation, migration, and invasion. Furthermore, CCL2 increased MAP3K19 expression by activating the MEK/ERK pathway. Thus, CCL2 is a vital molecular participant in ovarian cancer progression and may be a promising therapeutic target for ovarian cancer.

## 2. Results

### 2.1. Exogenous CCL2 Promoted the Migration and Invasion of Ovarian Cancer Cells

**Figure 1:**
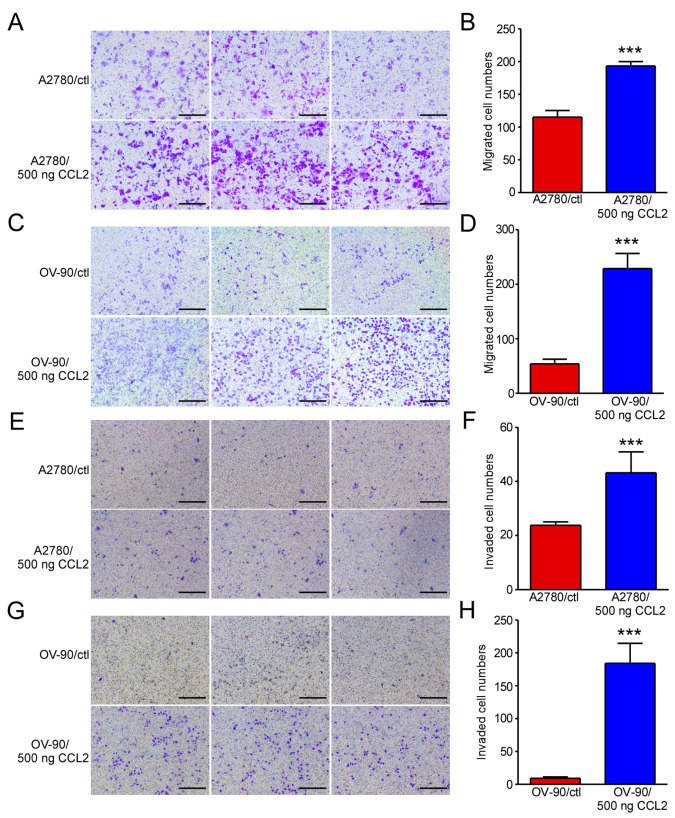
Exogenous CCL2 promoted the migration and invasion of A2780 and OV-90 cells. (**A**) Representative photographs of the migration ability of the A2780 cells treated with or without 500 ng CCL2 (n = 3). (**B**) Statistical analysis of (**A**). (**C**) Representative photographs of the migration ability of the OV-90 cells treated with or without 500 ng CCL2 (n = 3). (**D**) Statistical analysis of (**C**). (**E**) Representative photographs of the invasion ability of the A2780 cells treated with or without 500 ng CCL2 (n = 3). (**F**) Statistical analysis of (**E**). (**G**) Representative photographs of the invasion ability of the OV-90 cells treated with or without 500 ng CCL2 (n = 3). (**H**) Statistical analysis of (**G**). Scale bars: 50 μm. *** *p* < 0.001.

Our previous study demonstrated that exogenous CCL2 promotes ovarian cancer cell proliferation by stimulating ERK signaling and regulating the JUN, RELB, and NF-κB2 expressions [24]. We hypothesized that CCL2 plays an important role in ovarian cancer progression. In this work, we evaluated the vital role of CCL2 in ovarian cancer and explored the mechanism of CCL2 in the development of ovarian cancer. In the present study, cell migration and invasion assays were performed to determine the metastatic ability of CCL2 in ovarian cancer. After reviewing the literature, we selected 100–1000 ng CCL2 for the pre-experiment [25]. We tested the effects of different concentrations of CCL2 on the proliferation of A2780 cells and found that 500 ng had the best effect (Supplementary Figure S1). We selected this concentration for the cell migration and invasion experiments. A2780 and OV-90 cells were treated with 500 ng/mL CCL2 for 48 h. The CCL2-incubated A2780 cells demonstrated a higher migration capability than the cells in the negative control (Figure 1A,B). Similar results were observed in the OV-90 cells (Figure 1C,D). The CCL2-incubated A2780 and OV-90 cells demonstrated a higher invasion capability than the cells in the control group (Figure 1E–H). The results indicated that exogenous CCL2 could promote A2780 and OV-90 cell migration and invasion in vitro.

### 2.2. CCL2 Overexpression Promoted Ovarian Cancer Cell Proliferation, Migration, and Invasion

**Figure 2:**
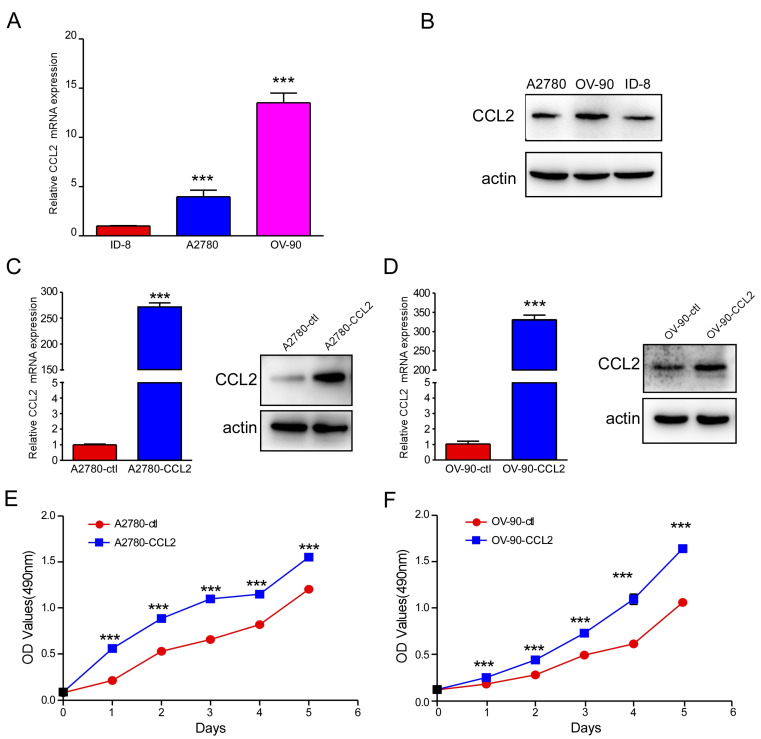
CCL2 overexpression promoted ovarian cancer cell proliferation. (**A**) qRT-PCR analysis exhibited CCL2 expression in different ovarian cancer cell lines. (**B**) CCL2 and actin were detected using Western blotting in different ovarian cancer cell lines. (**C**) CCL2 expressions were detected in the A2780 and CCL2-overexpressing A2780 cells using qRT-PCR and Western blotting. (**D**) qRT-PCR and Western blotting analysis exhibited the CCL2 mRNA and protein levels in the OV-90 and CCL2-overexpressing OV-90 cells. (**E**) CCL2 overexpression promoted A2780 cell proliferation. (**F**) CCL2 overexpression promoted OV-90 cell proliferation. *** *p* < 0.001.

**Figure 3:**
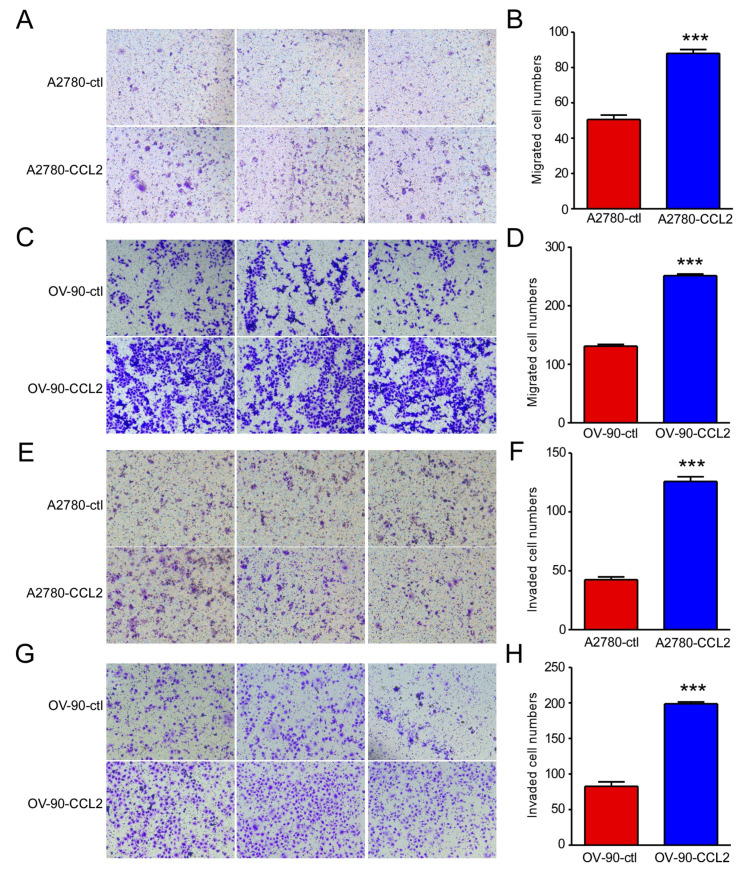
CCL2 overexpression promoted the migration and invasion of ovarian cancer cells. (**A**) Representative photographs of the migration ability of the A2780 and CCL2-overexpressing A2780 cells (n = 3). (**B**) Statistical analysis of (**A**). (**C**) Representative photographs of the migration ability of the OV-90 and CCL2-overexpressing OV-90 cells (n = 3). (**D**) Statistical analysis of (**C**). (**E**) Representative photographs of the invasion ability of the A2780 and CCL2-overexpressing A2780 cells (n = 3). (**F**) Statistical analysis of (**E**). (**G**) Representative photographs of the invasion ability of the OV-90 and CCL2-overexpressing OV-90 cells (n = 3). (**H**) Statistical analysis of (**G**). Scale bars: 50 μm. *** *p* < 0.001.

CCL2 expression was observed in ID-8, A2780, and OV-90 cells (Figure 2A,B). To further explore the role of CCL2 in ovarian cancer, it was overexpressed in A2780 and OV-90 cells. The overexpression efficiencies were examined using qRT-PCR and Western blotting. The mRNA and protein levels of CCL2 were upregulated in the CCL2-overexpressing A2780 and OV-90 cells, as expected, as shown in Figure 2C,D. Furthermore, an MTT assay was performed to determine the effect of CCL2 overexpression on A2780 and OV-90 cell proliferation. The CCL2 overexpression significantly promoted A2780 and OV-90 cell proliferation (Figure 2E,F). Transwell assays were performed to determine the CCL2 function in CCL2-overexpressing A2780 and OV-90 cells. The CCL2-overexpressing A2780 and OV-90 cells presented higher migration rates (Figure 3A–D). The CCL2-overexpressing A2780 and OV-90 cells demonstrated a higher invasion capability than the cells in the control group (Figure 3E–H). Taken together, the CCL2 overexpression promoted the proliferation, migration, and invasion of A2780 and OV-90 cells.

### 2.3. CCL2 Knockout Inhibited Ovarian Cancer Cell Proliferation, Migration, and Invasion

**Figure 4:**
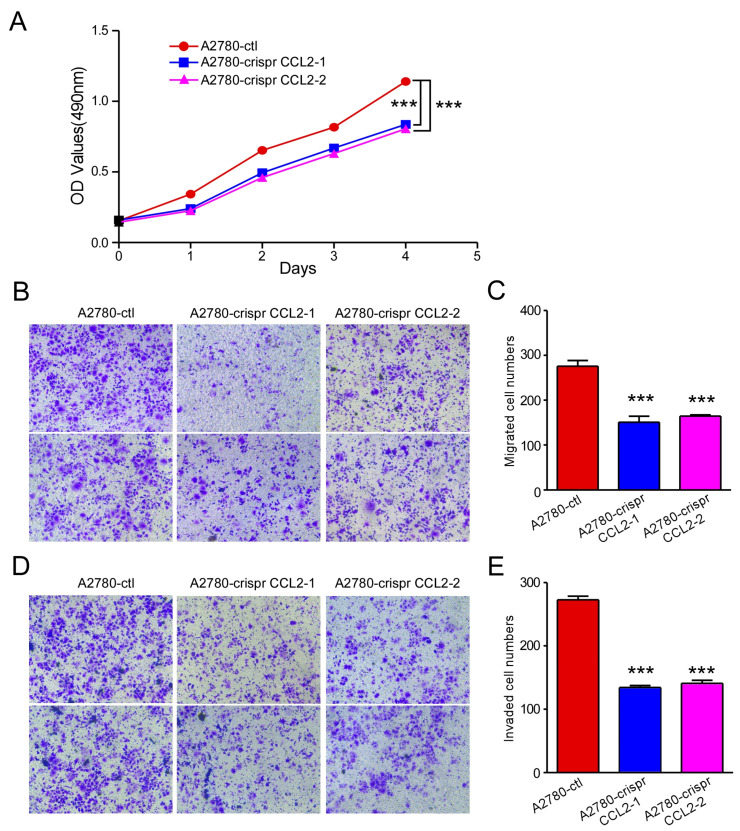
CCL2 knockout inhibited ovarian cancer cell proliferation, migration, and invasion. (**A**) CCL2 knockout using Crispr/Cas9 inhibited A2780 cell proliferation. (**B**) Representative photographs of the migration ability of the A2780 and CCL2-knockout A2780 cells (n = 3). (**C**) Statistical analysis of (**B**). (**D**) Representative photographs of the invasion ability of the A2780 and CCL2-knockout A2780 cells (n = 3). (**E**) Statistical analysis of (**D**). Scale bars: 50 μm. *** *p* < 0.001.

The function of CCL2 knockout in inhibiting ovarian cancer cell proliferation, migration, and invasion was investigated further to confirm the biological roles of CCL2 in ovarian cancer. The CRISPR/Cas9 assay was performed to stably knockout CCL2 in A2780 cells. Three sgRNA targeting exons 1 and 2 of CCL2 were designed, and recombinant PX-459-sgCCL2 plasmids were generated. PX-459-sgCCL2-1 and PX-459-sgCCL2-2 were transfected with A2780 to generate the Crispr CCL2-1 cell line, whereas PX-459-sgCCL2-1 and PX-459-sgCCL2-3 were transfected with A2780 to generate the Crispr CCL2-2 cell line. Furthermore, puromycin was used to select the stably transfected cells. The A2780 cells with stably knocked CCL2 and their control cells were screened. The CCL2 knockout led to a significant decrease in the A2780 cell proliferation (Figure 4A). Furthermore, the CCL2 knockout A2780 cells demonstrated lower migration (Figure 4B,C) and invasion (Figure 4D,E) capabilities than the cells in the control group. Thus, CCL2 knockout inhibited ovarian cancer proliferation, migration, and invasion.

### 2.4. MAP3K19 Was the Key Target for CCL2 in Regulating Ovarian Cancer Progression

**Figure 5:**
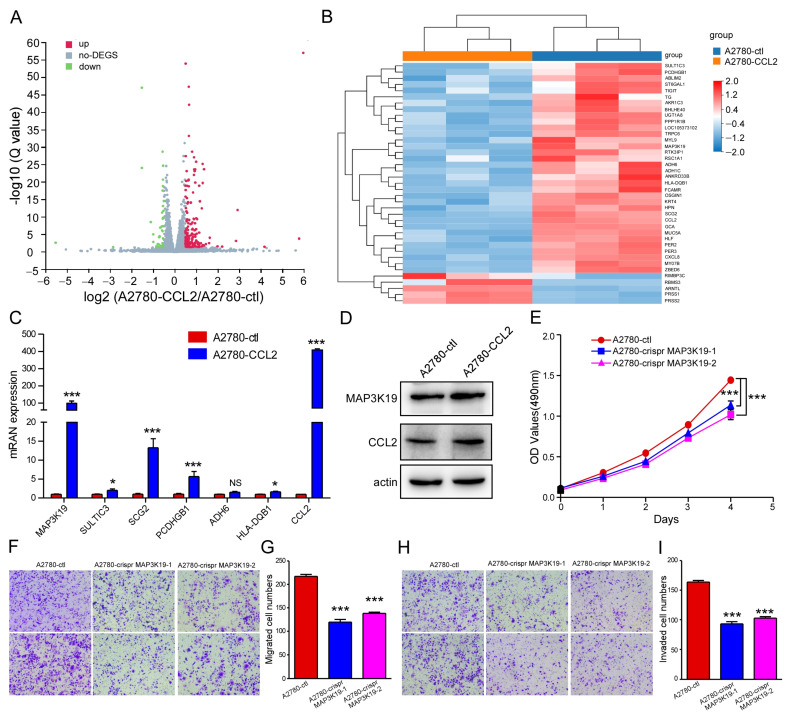
MAP3K19 was the key target for CCL2 in regulating ovarian cancer progression. (**A**) Volcano plot showing the differentially expressed genes in the CCL2-overexpressing A2780 cells compared with the A2780 cells. Upregulated transcription is depicted in red and downregulated transcription is depicted in green. (**B**) Heat map illustrating the genes with log_2_(fold change) ≥ 1 in the CCL2-overexpressing A2780 cells compared with the A2780 cells. Upregulated and downregulated transcription are depicted in red and blue, respectively. (**C**) qRT-PCR analysis of MAP3K19, SULTIC3, SCG2, PCDHGB1, ADH6, HLA-DQB1, and CCL2 expressions in the A2780 and CCL2-overexpressing A2780 cells. (**D**) Protein levels of MAP3K19, CCL2, and actin in the A2780 and CCL2-overexpressing A2780 cells, as found using Western blotting. (**E**) MAP3K19 knockout using Crispr/Cas9 inhibited A2780 cell proliferation. (**F**) Representative photographs of the migration ability of the A2780 and MAP3K19-knockout A2780 cells (n = 3). (**G**) Statistical analysis of (**F**). (**H**) Representative photographs of the invasion ability of the A2780 and MAP3K19-knockout A2780 cells (n = 3). (**I**) Statistical analysis of (**H**). Scale bars: 50 μm. NS means no statistical significance, * *p* < 0.05, *** *p* < 0.001.

A2780 control cells and CCL2-overexpressing A2780 cells were used to perform transcriptome sequencing. Overall, 16,584 genes were found expressed in the A2780 cells. A total of 215 genes underwent statistically significant changes in the CCL2-overexpressing A2780 cells when the genes log2 fold change ≥ 0.5. The volcano plot exhibits the upregulation of 178 and downregulation of 37 of these genes (Figure 5A). When the log2 fold change in the genes was ≥1, 34 were upregulated and 5 were downregulated. The most siginificantly upregulated genes were CCL2 (log2 fold change = 5.95) and MAP3K19 (log2 fold change = 5.78). Additionally, the five most significantly upregulated genes were SULTIC3 (log2 fold change = 4.17), SCG2 (log2 fold change = 2.93), PCDHGB1 (log2 fold change = 2.85), ADH6 (log2 fold change = 2.2), and HLA-DQB1 (log2 fold change = 1.99). Furthermore, MAP3K19, SULTIC3, SCG2, PCDHGB1, ADH6, and HLA-DQB1 expressions were verified using qRT-PCR. MAP3K19, SULTIC3, SCG2, PCDHGB1, and HLA-DQB1 expressions were upregulated in the CCL2-overexpressing A2780 cells, whereas ADH6 exhibited no change in expression (Figure 5C). The MAP3K19 protein level was elevated in the CCL2-overexpressing A2780 cells (Figure 5D).

### 2.5. MAP3K19 Knockout Inhibited Ovarian Cancer Cell Proliferation, Migration, and Invasion

To explore the biological roles of MAP3K19 in the proliferation, migration, and invasion of ovarian cancer cells, we subsequently knocked out MAP3K19 in the A2780 cells. Three MAP3K19 sgRNAs were designed and the recombinant PX-459-sg MAP3K19 plasmids were generated. PX-459-sgMAP3K19-1 and PX-459-sgMAP3K19-2 were transfected with A2780 to generate the Crispr MAP3K19-1 cell line, whereas PX-459-sgMAP3K19-1 and PX-459-sgMAP3K19-3 were transfected with A2780 to generate the Crispr MAP3K19-2 cell line. Furthermore, puromycin was used to select stably transfected cells. MAP3K19 knockout led to a significant decrease in the A2780 cell proliferation (Figure 5E). Furthermore, the MAP3K19-knockout A2780 cells demonstrated lower migration (Figure 5F,G) and invasion (Figure 5H,I) capabilities than the cells in the control group. Thus, MAP3K19 knockout inhibited the proliferation, migration, and invasion of ovarian cancer cells.

### 2.6. CCL2 Promoted MAP3K19 Expression by Activating the MEK/ERK Pathway

**Figure 6:**
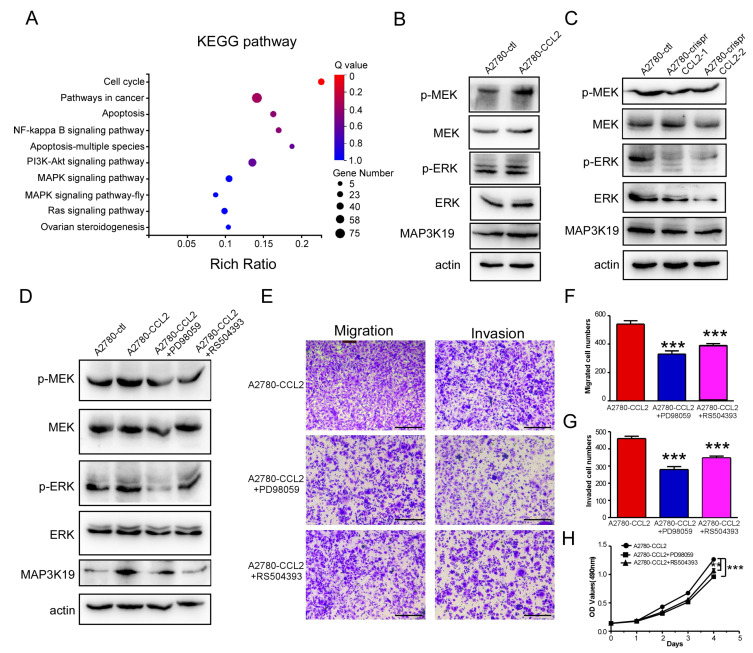
CCL2 promoted MAP3K19 expression through the MEK/ERK pathway. (**A**) Ten representative signaling pathways were upregulated by CCL2 in the A2780 cells. (**B**) The protein levels of *p*-MEK, MEK, p-ERK, ERK, MAP3K19, and actin were analyzed using Western blotting in the A2780 and CCL2-overexpressing A2780 cells. (**C**) The expressions of phosphorylated MEK, phosphorylated ERK, MAP3K19, MEK, ERK, and actin were analyzed using Western blotting in the A2780 and CCL2-knockout A2780 cells. (**D**) The expressions of phosphorylated MEK, phosphorylated ERK, MAP3K19, MEK, ERK, and actin were analyzed using Western blotting in the following cells: A2780, CCL2-overexpressing A2780, CCL2-overexpressing A2780 pretreated with PD98059, and CCL2-overexpressing A2780 pretreated with RS504393. (**E**) Representative photographs of the migration (left) and invasion (right) abilities of the following cells: CCL2-overexpressing A2780, CCL2-overexpressing A2780 pretreated with PD98059, and CCL2-overexpressing A2780 pretreated with RS504393 (n = 3). (**F**) Statistical analysis of (left side of (**F**)). (**G**) Statistical analysis of (right side of (**F**)). (**H**) PD98059 and RS504393 inhibited the A2780-CCL2 cell proliferation. Scale bars: 50 μm. ** *p* < 0.01, *** *p* < 0.001.

CCL2 significantly promoted the proliferation, migration, and invasion of ovarian cancer cells by targeting MAP3K19. CCL2 was found to have selectively altered the activity of 10 representative signaling pathways in A2780 cells using transcriptome analysis (Figure 6A). NF-κB, AKT, P38, and ERK signaling pathways were identified as the key regulators in ovarian cancer progression [27,28,29]. The CCL2-overexpressing A2780 cells exhibited higher levels of phosphorylated MEK, phosphorylated ERK, and MAP3K19 than the control group (Figure 6B). Additionally, the CCL2-overexpressing A2780 cells exhibited a decrease in P65, P38, and AKT signaling (Supplementary Figure S2). Moreover, the CCL2-knockout A2780 cells exhibited lower phosphorylated MEK, phosphorylated ERK, and MAP3K19 levels than the control group (Figure 6C). To further determine whether CCL2 promoted MAP3K19 expression by regulating the MEK/ERK pathway, the MEK inhibitor PD98059 was added to the CCL2-overexpressing A2780 cells. Pretreatment with PD98059 decreased the phosphorylated MEK and ERK levels and the protein level of MAP3K19 in the CCL2-overexpressing A2780 cells (line 3 of Figure 6D). CCL2 mainly mediated its functions by binding to its receptor CCR2 and triggering a series of signal transduction reactions. Then, the CCR2 inhibitor RS504393 was added to the CCL2-overexpressing A2780 cells to block the binding of CCL2 to CCR2. The CCL2-overexpressing A2780 cells exhibited increased levels of phosphorylated MEK, phosphorylated ERK, and MAP3K19, whereas this phenotype could be impaired by treatment with RS504393 (Figure 6D). The CCL2-overexpressing A2780 cells pretreatment with PD98059 or RS504393 demonstrated lower migration (the left sides of Figure 6E,F) and invasion (the right sides of Figure 6E,G) capabilities than the cells in the control group. Furthermore, the CCL2-overexpressing A2780 cells pretreatment with PD98059 or RS504393 led to a significant decrease in cell proliferation (Figure 6H). These results indicated that CCL2 promoted MAP3K19 expression through the MEK/ERK signaling pathway.

## 3. Discussion

Several studies indicated the active role of cytokines in the progression of gynecological tumors. The serum CCL2 level in patients with primary ovarian cancer is higher than that in patients with benign ovarian cysts and healthy people. Additionally, CCL2 expression is upregulated in MA-148 (an ovarian cancer cell line) under treatment with paclitaxel and carboplatin. CCL2 stimulates ovarian cancer progression by enhancing angiogenesis [22,23]. Furthermore, exogenous CCL2 stimulated the migration and adhesion of SKOV-3 cells, which could be reduced by adding a CCR2 antagonist [25,30]. CCL2 from mesenchymal stromal cells acts on ovarian cancer cells and induces IL-6 secretion [31]. Cancer-associated mesothelial cells enhance epithelial ovarian cancer cell invasion by generating CCL2 through the P38 pathway. CCL2 inhibition by neutralizing antibodies reduced epithelial ovarian cancer cell invasion [26]. However, the underlying mechanisms and possible roles of CCL2 in regulating ovarian cancer remain unclear. Our previous study reported that the recombinant CCL2 protein promoted ovarian cancer cell activity [24]. HGSOC is the most lethal gynecologic cancer and the fifth most common cancer among women worldwide. Studies demonstrated that GATA3 is associated with poor prognosis in patients with HGSOC. Previous studies indicated that overexpression of GATA3 in HGSOC cells promotes the expression of p-ERK1/2 and the activation of p-ERK1/2 suppresses TP53 [32]. TP53 is associated with macrophage infiltration in the tumor microenvironment (TME) [33]. B7-H3 (CD276) is involved in the progression of HGSOC through CCL2-CCR2-tumor-associated macrophages (TAMs) axis-mediated immunosuppression [34].

The present study exhibited that recombinant CCL2 protein promoted ovarian cancer cell migration and invasion. CCL2 overexpression promoted the proliferation, migration, and invasion of ovarian cancer cells. Additionally, CCL2 knockout in A2780 cells inhibited the biological activity of ovarian cancer cells. Transcriptome sequencing demonstrated that MAP3K19 was the key target for CCL2 in regulating ovarian cancer progression. Moreover, MAP3K19 knockout inhibited the biological activity of ovarian cancer cells. Transcriptome analysis demonstrated several pathways that were closely related to ovarian cancer development. Among these pathways, the MEK and ERK phosphorylation levels were elevated in the CCL2-overexpressing A2780 cells. CCL2 promoted MAP3K19 expression and was significantly inhibited in the CCL2-overexpressing A2780 cells pretreated with PD98059 and RS504393 (Figure 6D). Thus, CCL2 increased MAP3K19 expression by activating the MEK/ERK pathway.

MAP3K19 is also known as YSK-4, and the Ser/Thr kinase leads to the activation of MAPKs. Studies demonstrated that MAP3K19 expression is elevated in idiopathic pulmonary fibrosis (IPF) tissues. MAP3K19 inhibition by a siRNA inhibitor significantly attenuated pulmonary fibrosis in a bleomycin-induced murine model [35]. MAP3K19 inhibition by siRNA also attenuated the profibrotic activity of cultured fibroblasts in an adoptive transfer mouse model of pulmonary fibrosis [36]. Thus, MAP3K19 may be a promising therapeutic target for idiopathic pulmonary fibrosis. Moreover, MAP3K19 expression is increased in chronic obstructive pulmonary disease [37]. Van T. et al. exhibited that MAP3K19 promotes the extracellular regulation of protein kinase and c-jun N-terminal kinase in several lung cancer cells. MAP3K19 inhibition is crucial for the survival of Kras mutant cancers, which inactivates the extracellular regulated protein kinase and c-jun N-terminal kinase pathways [38]. The present study exhibited that MAP3K19 (log2 fold change = 5.78) was the most significantly upregulated gene in the CCL2-overexpressing A2780 cells. Additionally, the results were confirmed using qRT-PCR and Western blotting. MAP3K19 knockout inhibited the biological functions of ovarian cancer cells, and thus, it may be the critical target of CCL2 in the regulation of ovarian cancer development.

## 4. Materials and Methods

### 4.1. Cell Lines and Cell Culture

Human epithelial ovarian cancer cell lines, namely, OV-90 (MZ-2097, MINGZHOUBIO, Ningbo, China) and A2780 (CBP60283, Cobioer Biosciences, Nanjing, China), were stored in our laboratory. A2780—ovarian endometrioid adenocarcinoma. OV-90—High-grade serous ovarian cancer derived from metastatic site: ascites. Mouse epithelial ovarian cancer cell line ID-8 was stored in our laboratory. ID-8, OV-90, and A2780 cells were cultured in Dulbecco’s modified Eagle’s medium (DMEM, Gibco, C11995500BT) in an incubator with 5% CO_2_ at 37 °C. The complete medium contained 10% fetal bovine serum (FBS, Biological Industries, Israel, C04001500) and 1% penicillin–streptomycin (Cytiva, Logan, UT, USA, SV30010).

### 4.2. Generation of CCL2-Overexpressing Cell Lines

The human CCL2 gene was inserted into the pCDH-CMV-MCS-EF1-CopGFP vector to generate a recombinant pCDH-CCL2 plasmid. A2780 cells were cultured in a 6-well plate. When cells had grown to 70–90%, they were transfected with pCDH-CMV-MCS-EF1-CopGFP or pCDH-CCL2 by using Lipofectamine 3000 according to the instructions. Puromycin was used to select stably transfected cells [39]. The overexpression efficacies of CCL2 were evaluated using Western blotting and real-time quantitative reverse transcription-polymerase chain reaction (qRT-PCR).

### 4.3. Generation of KO Cell Lines Using CRISPR/Cas9

Guide RNAs (sgRNAs) were designed with the online CRISPR Guide RNA design tool, namely, Benchling (https://www.benchling.com/crispr/, (accessed on 1 January 2023)). Primer sequences for CCL2 and MAP3K19 sgRNA are listed in Supplementary Table S1. SgRNAs were inserted into the pSpCas9 (BB)-2A-puro (PX459) plasmid to generate recombinant plasmids. PX-459-sgCCL2-1 and PX-459-sgCCL2-2 were transfected with A2780 to generate the Crispr CCL2-1 cell line. PX-459-sgCCL2-1 and PX-459-sgCCL2-3 were transfected with A2780 to generate the Crispr CCL2-2 cell line. PX-459-sgMAP3K19-1 and PX-459-sgMAP3K19-2 were transfected with A2780 to generate the Crispr MAP3K19-1 cell line. PX-459-sgMAP3K19-1 and PX-459-sgMAP3K19-3 were transfected with A2780 to generate the Crispr MAP3K19-2 cell line. Furthermore, puromycin was used to select stably transfected cells [40].

### 4.4. Transcriptome Sequencing and Analysis

A2780 cells and CCL2-overexpressing A2780 cells were used to perform transcriptome sequencing. Transcriptome sequencing and analysis were performed by the Beijing Genomics Institution. RNA-Seq data reported in this study can be accessed at the National Center for Biotechnology Information (NCBI) with the accession number PRJNA800238.

### 4.5. MTT Assays

Cell proliferation rates were measured using a 3-(4,5-dimethyl-2-thiazolyl)-2,5-diphenyl-2-H-tetrazolium bromide (MTT, Beyotime, ST316) assay. The cells were treated in DMEM without FBS for 12 h. Then, 3 × 10^3^ cells were cultured in a 96-well plate for 0, 24, 48, and 72 h, followed by the addition of 10 μL of MTT solution (5 mg/mL) for 3 h. The cell medium was then extracted from each well. Finally, 100 mL of dimethyl sulfoxide was added to the wells and the OD490 was measured [41].

### 4.6. Cell Migration and Invasion Assays

A2780 and OV-90 cells were treated in an FBS-free medium for 12 h. A2780 and OV-90 cells were counted, and 5 × 10^4^ cells were cultured in the upper chamber for the cell migration assay. Then, 5 × 10^4^ cells were cultured in the Matrigel-coated upper chamber for the invasion assay. A total of 500 µL of complete medium was added to the lower chamber. After being incubated for 48 h, the cells were removed from the upper chamber. These cells migrated onto the lower membrane surface, where they were fixed with 4% formaldehyde (Biosharp, Hefei, China, BL539A) and stained with 10% Giemsa stain (Biosharp, BL802A). The migrated cells were quantified by counting the stained cells under a microscope [42]. Each experiment was repeated in at least three wells. For the exogenous CCL2-stimulated migration and invasion assays, 500 ng CCL2 was added to the lower chamber.

### 4.7. Inhibitor Treatment Assays

CCL2-overexpressing A2780 cells were pretreated with 20 μm/mL PD98059 or 20 um/mL RS504393 for 4 h to explore the influence of the MEK inhibitor PD98059 and CCR2 inhibitor RS504393 on CCL2-induced signaling transduction. Then, the cells were cultured for another 48 h. The protein was extracted and detected using Western blotting [43].

### 4.8. Quantitative Real-Time PCR

Total RNA was extracted using TRIzol (Zomanbio, Beijing, China, ZP401) according to the manufacturer’s instructions. The ReverTra Ace^®^ qRT-PCR kit was used with 1 mg total RNA to synthesize cDNA. The qRT-PCR assay was performed with 40 cycles of denaturation at 94 °C (30 s), annealing at 58 °C (30 s), and extension at 72 °C (1 min) by using the SYBR Premix Ex Taq. The primer sequences of CCL2; MAP3K19; SULTIC3; secretogranin II (SCG2); protocadherin gamma subfamily B, 1 (PCDHGB1); alcohol dehydrogenase 6 (ADH6); major histocompatibility complex, class II, DQ beta 1 (HLA-DQB1); and glyceraldehyde-3-phosphate dehydrogenase (GAPDH) for qRT-PCR are presented in Supplementary Table S2. GAPDH expression was used for endogenous normalization.

### 4.9. Western Blotting

The protein was extracted using a radio-immunoprecipitation assay (RIPA) buffer with phenylmethylsulfonyl fluoride (PMSF) and phosphatase inhibitor. Total proteins were analyzed using 10% SDS-PAGE and transferred onto the polyvinylidene fluoride (PVDF) membranes. The membranes were blocked with 5% milk and probed with primary antibodies overnight at 4 °C. After being washed thrice with 1 × TBST, the blots were incubated with HRP-conjugated secondary antibodies for 2 h. Additionally, antibodies against CCL2 (Proteintech, Wuhan, China, 66272-1-Ig), MAP3K19 (Abbkine, ABP60945), p-MEK (Abbkine, ABP50356), p-ERK (Abbkine, ABP50531), MEK (Abbkine, Wuhan, China, ABP51772), ERK (Abbkine, ABM40224), and actin (Zomanbio, Beijing, China, ZN102A) were used at 1:2000 dilution. The signals were visualized using ECLTM Western blotting reagents [44].

### 4.10. Statistical Analysis

All results are presented as the mean ± standard deviation. Comparisons between paired groups were made using Student’s *t*-test. Statistical analyses were conducted using GraphPad Prism 5.0. A *p*-value < 0.05 was considered statistically significant.

## 5. Conclusions

**Figure 7:**
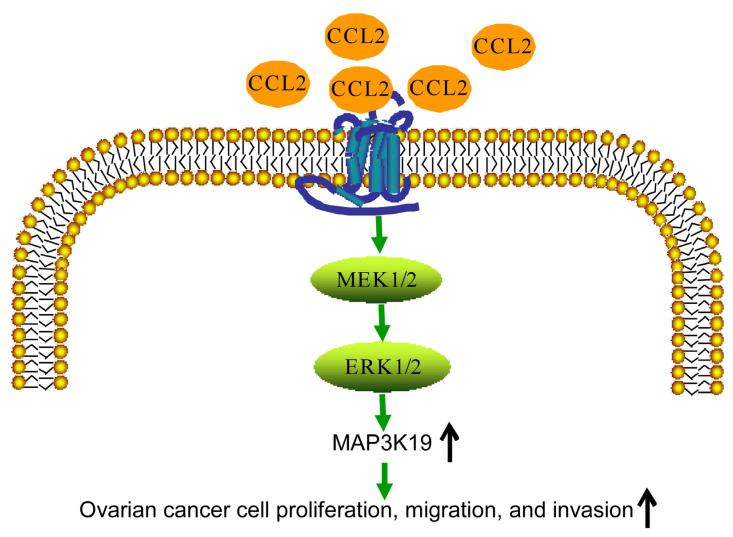
Schematic model. CCL2 promotes ovarian cancer progression through the MEK/ERK/MAPP3K19 signaling pathway.

The present study demonstrated that exogenous CCL2 and CCL2 overexpression promoted the biological functions of ovarian cancer cells, whereas CCL2 knockout inhibited ovarian cancer cell activity, migration, and invasion. MAP3K19 was the key target for CCL2 in the regulation of ovarian cancer progression. Moreover, MAP3K19 knockout inhibited the biological functions of ovarian cancer cells. Additionally, CCL2 increased MAP3K19 expression by targeting the MEK/ERK signaling pathway (Figure 7). Thus, the present study uncovered the association between CCL2 and ovarian cancer, suggesting that CCL2 may be a promising therapeutic target in ovarian cancer. However, clinical data and animal experiments are required to understand the role of CCL2 in ovarian cancer development in detail.

## Data Availability

The data that support the findings of this study are available from the corresponding author upon reasonable request.

## Supplementary Materials

The following supporting information can be downloaded from https://www.mdpi.com/article/10.3390/ijms241310652/s1.

## Abbreviations

CCL2: CC chemokine 2; MCP-1: monocyte chemotactic protein-1; CCR2: Chemokines (C-C motif) receptor 2; MAPK: mitogen-activated protein kinase; MEK: mitogen-activated protein kinase kinase; ERK: extracellular signal-regulated kinase; AKT: protein kinase B; NF-κB: nuclear factor-kappaB; MAP3K19: Mitogen-activated protein three kinase 19; IL-6: interleukin-6; MTT: 3-(4,5-dimethyl-2-thiazolyl)-2,5-diphenyl-2-H-tetrazolium bromide; SCG2: secretogranin II; PCDHGB1: protocadherin gamma subfamily B, 1; ADH6: alcohol dehydrogenase 6; HLA-DQB1: major histocompatibility complex, class II, DQ beta 1.
