# Supplementary material for: CC Chemokine 2 Promotes Ovarian Cancer Progression through the MEK/ERK/MAP3K19 Signaling Pathway
Source: Int J Mol Sci. 2023 Jun 26;24(13):10652. doi: 10.3390/ijms241310652 (PMC10341728; doi:10.3390/ijms241310652)
Supplement: Supplementary file 1 [file ijms-24-10652-s001.zip › ijms-2438618-supplementary.pdf]

Supplementary Materials

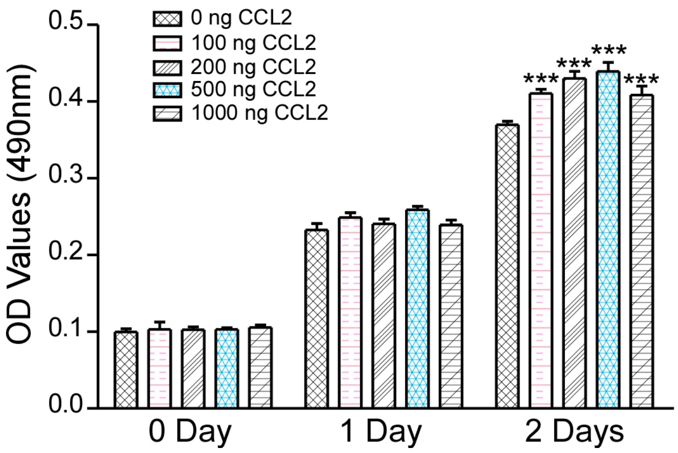

Supplementary Figure S1. The function of CCL2 on the proliferation of A2780 cells at different concentrations. ****P* means *P* <0.001.

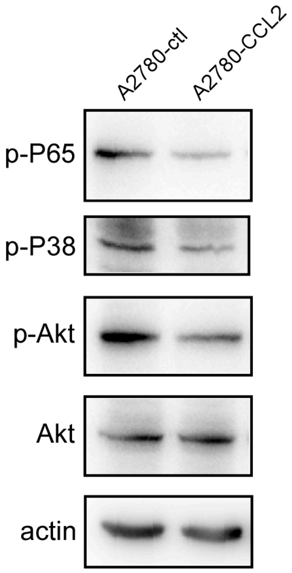

Supplementary Figure S2. The expressions of phosphorylated P65, phosphorylated P38, phosphorylated Akt, Akt, and actin were analysed by Western blotting in A2780 and CCL2-knockout A2780 cells.

Supplementary Table S1. Primer sequences for plasmid construction

CCL2 sgRNA-1	Fw	CACCGAGCCACCTTCATTCCCCAA
	Rv	AAACTTGGGGAATGAAGGTGGCTC
CCL2 sgRNA-2	Fw	CACCGTAGGAAGATCTCAGTGCAG
	Rv	AAACCTGCACTGAGATCTTCCTAC
CCL2 sgRNA-3	Fw	CACCGTTATAACAGCAGGTGACTG
	Rv	AAACCAGTCACCTGCTGTTATAAC
MAP3K19 sgRNA-1	Fw	CACCGGACTGCAGTCATTCCACAC
	Rv	AAACGTGTGGAATGACTGCAGTCC
MAP3K19 sgRNA-2	Fw	CACCGCAGTGGTGGTAGACAGGAC
	Rv	AAACGTCCTGTCTACCACCACTGC
MAP3K19 sgRNA-3	Fw	CACCGCCTCTCATATGAAGTACAG
	Rv	AAACCTGTACTTCATATGAGAGGC

Supplementary Table S2. Primer sequences for qRT-PCR analysis

CCL2	Fw	CAGCCAGATGCAATCAATGCC
	Rv	TGGAATCCTGAACCCACTTCT
MAP3K19	Fw	ACCATGCGGCCCTTAGTTTT
	Rv	ATGCTTCGGCCACTGTACTTC
SULTIC3	Fw	GGCTTCCTTTATGCCTGATCC
	Rv	CAGGACCCGCCAACAACTTT
SCG2	Fw	ACCAGACCTCAGGTTGGAAAA
	Rv	AAGTGGCTTTCATCGCCATTT
PCDHGB1	Fw	GTGAACGGTAGGATAGATCGAGA
	Rv	TCAGTGAATTGCCTTCCACATC
ADH6	Fw	ACAGGCCAAGTCATCAGATGC
	Rv	CCACAACCTTTATGCGAACTTCC
HLA-DQB1	Fw	GCGGGATCTTGCAGAGGAG
	Rv	ACTTTGATCTGGCCTGGATAGAA
GAPDH	Fw	ACAACTTTGGTATCGTGGAAGG
	Rv	GCCATCACGCCACAGTTTC
